# BSafe-360: An All-in-One Naturalistic Cycling Data Collection Tool

**DOI:** 10.3390/s23146471

**Published:** 2023-07-17

**Authors:** Suzana Duran Bernardes, Kaan Ozbay

**Affiliations:** C2SMART Center (Tier 1 UTC Funded by USDOT), Department of Civil and Urban Engineering, New York University, 6 MetroTech Center 4th Floor, Brooklyn, NY 11201, USA; kaan.ozbay@nyu.edu

**Keywords:** bicycle safety, data acquisition system, lateral passing distance, naturalistic cycling data, sensing

## Abstract

The popularity of bicycles as a mode of transportation has been steadily increasing. However, concerns about cyclist safety persist due to a need for comprehensive data. This data scarcity hinders accurate assessment of bicycle safety and identification of factors that contribute to the occurrence and severity of bicycle collisions in urban environments. This paper presents the development of the BSafe-360, a novel multi-sensor device designed as a data acquisition system (DAS) for collecting naturalistic cycling data, which provides a high granularity of cyclist behavior and interactions with other road users. For the hardware component, the BSafe-360 utilizes a Raspberry Pi microcomputer, a Global Positioning System (GPS) antenna and receiver, two ultrasonic sensors, an inertial measurement unit (IMU), and a real-time clock (RTC), which are all housed within a customized bicycle phone case. To handle the software aspect, BSafe-360 has two Python scripts that manage data processing and storage in both local and online databases. To demonstrate the capabilities of the device, we conducted a proof of concept experiment, collecting data for seven hours. In addition to utilizing the BSafe-360, we included data from CCTV and weather information in the data analysis step for verifying the occurrence of critical events, ensuring comprehensive coverage of all relevant information. The combination of sensors within a single device enables the collection of crucial data for bicycle safety studies, including bicycle trajectory, lateral passing distance (LPD), and cyclist behavior. Our findings show that the BSafe-360 is a promising tool for collecting naturalistic cycling data, facilitating a deeper understanding of bicycle safety and improving it. By effectively improving bicycle safety, numerous benefits can be realized, including the potential to reduce bicycle injuries and fatalities to zero in the near future.

## 1. Introduction

Bicycle safety is a key aspect of sustainable and equitable transportation in urban environments. It has become a prominent concern for researchers in recent years, especially since the changes in society’s priorities caused by the COVID-19 pandemic and the economic crisis. Since 2012, cities around the United States (U.S.) have adopted the Vision Zero program, the main goal of which is to achieve zero traffic fatalities and injuries. The program originated in Sweden and has been successfully implemented in various European cities, bringing the number of cyclists injured or killed to nearly zero in some countries [1]. However, bicycle fatalities are still on the rise in the US [2]. This scenario raises questions about what factors and guidelines truly contribute to improving bicycle safety in urban environments.

One particular concern is the lateral passing distance (LPD) between cyclists and motor vehicles. This distance can significantly affect cyclists’ safety, as it determines the amount of space drivers must allow when overtaking cyclists. For example, 29% of the crashes involving motor vehicles and bicycles were due to these vehicles traveling adjacent to each other in New York City (NYC) [3]. However, current data on LPD are limited. They are often based on the drivers’ perspective because of the wide availability of state-of-the-art data acquisition systems (DAS) for motorized vehicles (e.g., cars and trucks), especially for connected and autonomous vehicles, making it difficult to assess current bicycle safety measure effectiveness accurately.

Fortunately, the advancement and spread of technologies in sensing, the Internet of Things (IoT), and Big Data allowed the improvement of data collection in various areas of study, including bicycle safety. Researchers have begun to turn especially to smartphone data and the development of instrumented bicycles (IBs), that is, bicycles equipped with two or more sensors, to function as bicycle-specific DAS [4,5,6,7,8]. The main advantage of these new DAS is that they allow the collection of naturalistic cycling data, which provides detailed data about cycling behavior and infrastructure. Naturalistic cycling data correspond to cyclists’ real-world behaviors and environmental interactions collected without any interference from researchers [9]. However, the current scenario of bicycle DASs for the collection of naturalistic cycling data is still in its early stages. It has room for improvement with respect to its reliability, precision, availability, size, monetary and time costs, and scalability. Such improvements in DASs to collect naturalistic cycling data can help answer long-standing research questions, such as what factors lead to higher collisions, injuries, and fatalities involving bicycles, or what is the optimal threshold for the safe LPD drivers should maintain from cyclists when overtaking them.

Therefore, this paper addresses the research question of how to successfully collect naturalistic cycling data to effectively measure safety risks using emerging technologies by introducing a novel alternative DAS called BSafe-360. The main goal of this study is to introduce and evaluate the efficacy of BSafe-360 as an all-in-one platform for collecting naturalistic cycling data. While various methods have been employed in the past for collecting such data, they often suffer from issues of data synchronization, interpolation, and cost. In contrast, BSafe-360 overcomes these limitations by integrating multiple sensors into a single DAS, which includes a Global Positioning System (GPS) antenna and a receiver, two ultrasonic sensors, an inertial measurement unit (IMU), and a real-time clock (RTC) enclosed in a portable case, resulting in a more streamlined and efficient data collection process. By evaluating the feasibility and potential of the BSafe-360 system through a case study, this paper demonstrates its ability to gather critical information on cyclist behavior, trajectory, and LPDs. The ultimate goal is to provide an efficient and reliable way to collect naturalistic cycling data that can be used for safety studies, policy-making, and infrastructure design.

The next section (Section 2) presents a literature review of cycling data collection methods, focusing on their use for safety studies. Section 3 covers the details of the development of BSafe-360, including hardware and software components. Section 4 presents its implementation in a case study for proof of concept. Section 5 discusses the paper’s findings, contributions, and limitations based on lessons learned during the development of the device and on the results of the case study. Finally, Section 6 presents our main conclusions.

## 2. Literature Review

Bicycle safety is a crucial concern, particularly in urban areas where more people are opting for cycling as a means of transportation. Although there has been extensive research on specific issues such as collision severity and bicycle network, it is important to conduct further studies on bicycle safety to address a wider range of factors that contribute to improving bicycle safety [10]. One critical area of research that requires exploration is the collection of naturalistic cycling data, as this type of data can be crucial for advancing several niches of bicycle safety [11,12]. By gathering data in actual cycling conditions, researchers can gain a better understanding of the different factors that contribute to bicycle collision, injuries, and fatalities [13]. This method allows a more comprehensive and holistic approach to studying bicycle safety as it captures information on the interactions between cyclists, motorists, pedestrians, and the surrounding environment [14]. Furthermore, such data can help researchers define cyclist behaviors and comprehend the factors that influence cyclists’ decision-making process while on the road [15]. Finally, naturalistic cycling data can provide insights into how conditions of the road are associated with cyclist safety and comfort [16]. In recent times, technological advancements have made collecting naturalistic cycling data more feasible.

In the last two decades, several methods have been introduced for collecting naturalistic and non-naturalistic cycling data, including surveys and GPS [17], video footage [18,19], virtual reality [20], and other sensors (e.g., LiDAR and ultrasonic sensor)  [21,22], the latter being the use of commercial sensors installed on a bicycle (i.e., instrumented bicycles) [4,5,6,21] or combined in one device that can be mounted on bicycles [23,24,25].

The development of IBs and devices for the collection of naturalistic cycling data has brought important contributions to the field of naturalistic cycling, ranging from improving performance tracking to improving traffic safety. Some of the first instrumented bicycles were introduced by Joo and Oh (2013) [4] to monitor cycling infrastructure and by Dozza, Werneke, and Fernandez (2012) [5] and Dozza and Werneke (2014) [6] to identify factors that influence cyclist safety. As IBs depend on the installation of several sensors to the bicycle, the quantity of sensors that could be used is limited by the space available on the bicycle. The study budget could also limit the number of sensors used. Therefore, the number and type of sensors utilized to instrument a bicycle highly depend on the objective of the study, which hinders the application of the same IB to different studies. For example, while Joo and Oh (2013) [4] equipped a bicycle with a GPS computer and an IMU board, Dozza, Werneke, and Fernandez (2012) [5] and Dozza and Werneke (2014) [6] equipped a bicycle with six sensors (e.g., HMI, GPS, IMU, Brake Force sensor, Speed sensor, Start-Stop sensor), a wireless modem, and a camera. However, all authors combined the data acquired using a separate software, which required synchronization and interpolation of the data. In another study, Dozza, Rasch, and Boda (2017) [7] introduced the use of an RPi as data logger to integrate the data collected from an IMU, a speed sensor, and an angle sensor installed in a bicycle. The introduction of the RPi contributes to reducing the costs involved in performing data integration post-processing, but the configuration of the IB is still bulky and can interfere with how the volunteer would normally ride.

The most recent iterations of IBs include the adoption of more advanced sensing technologies such as LiDAR [7,26] and virtual reality [27]. The concept of IBs has also been adapted to other non-motorized vehicles, such as electric bicycles and e-scooters [14,28]. Pérez-Zuriaga et al. (2022) [28] equipped an e-scooter with two ultrasonic sensors, an IMU, a video camera GPS, and an RPi to collect LPD and the vibrations experienced by the e-scooter users when riding on a bicycle lane. A study by Ma et al. (2021) [29] used similar components but replaced the ultrasonic sensor with a LiDAR sensor. Although LiDAR improved the accuracy of distance measurements, it required a larger case and odd positioning on the e-scooter than the configuration suggested by Pérez-Zuriaga et al. (2022) [28]. However, most IBs and instrumented e-scooters result in sensors taking up a lot of space on the bicycle, which can interfere with the natural user behavior. Thus, other researchers started to look into alternatives to develop DAS that can be customized to support the same sensors as IBs but are portable and can be easily mounted and un-mounted on bicycles for collecting naturalistic cycling data, especially LPDs [23,24,25].

To our knowledge, the first efforts to produce an all-in-one device to provide an alternative to instrumenting a bicycle were made in 2019 with the first prototypes of MetreBox [23] and BSafe-360 (referred to as a portable multi-sensor platform back then) [24]. Both devices contained GPS and ultrasonic sensors and were enclosed by a customized 3D printed case. The main difference between the devices is that while the MetreBox used only one ultrasonic sensor and an Arduino microprocessor, the portable multi-sensor platform used two ultrasonic sensors, one on each side of the bicycle, and a Raspberry Pi 2 B microcomputer.

Since then, other researchers have attempted to create new versions of all-in-one devices. For instance, Henao et al. (2021) [25] developed an open-source naturalistic cycling data collection DAS (1M+) using an RPi, a time-to-flight sensor, a GPS, a camera, and a custom 3D printed case. Nolan et al. (2021) [30] introduced the PassBox, a DAS which is a combination of the all-in-one device with IBs approaches. The authors used a device composed of two commercial ultrasonic sensors and a GPS receiver connected to an Arduino board enclosed by a plastic project box. This device has the potential to become an all-in-one DAS. However, they also used two Garmin Virb X action cameras attached to other parts of the bicycle to obtain additional video, GPS, accelerometer, and gyroscope data, which is more similar to the IB approach. Finally, Rudolph et al. (2022) [31] implemented the use of the OpenBikeSensor for measuring LPD. The OpenBikeSensor is an all-in-one DAS with two ultrasonic sensors and a GPS receiver connected to a printed circuit board (PCB) created by their research team. The DAS also has a separate small monitor connected to the main board via external cables that allow for setting up internet connection, privacy zones, and user identification of vehicles overtaking the bicycle [32]. Table 1 summarizes the major studies that included details of the development and use case of novel DASs for bicycle and micromobility studies.

Although much progress has been made toward building reliable DASs for naturalistic cycling data collection to improve overall bicycle safety, researchers in the field agree that there is still a great deal of research to develop better DASs for collecting naturalistic cycling data [34]. For now, the practices involved in the development and usage of these DASs are still decentralized, which can lead to bias. The sensors used and their position on the bicycle vary according to the study location, objective, and assumptions. Therefore, it is especially important to improve these DASs when it comes to standardization of procedures, costs, data synchronization, and scalability.

## 3. Materials and Methods

**Figure 1:**
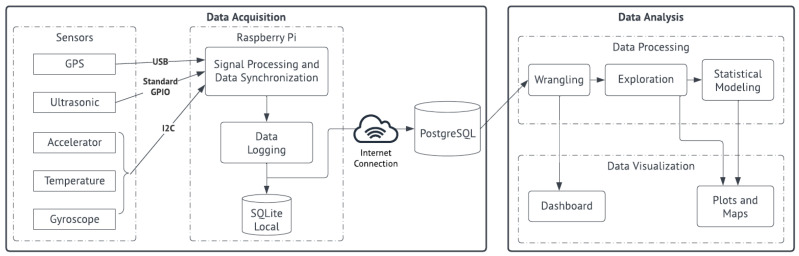
Detailed BSafe-360 platform architecture, including the depiction of components and steps of the DAS and the data analysis.

This section presents the processes involved in the development of BSafe-360. Figure 1 shows the architecture of the DAS and the steps taken during the data analysis stage, which are further discussed in the next sections.

### 3.1. Data Acquisition

#### 3.1.1. Hardware

To build the BSafe-360 device, we determined exactly what data we expected to obtain based on our main research question (i.e., how can we successfully collect naturalistic cycling data that can be used for bicycle safety studies). Expected data should include at least the bicycle trajectory and the distance from the bicycle to the lateral obstacles. Other information, such as acceleration, yaw, pitch, and roll of the bicycle, is also relevant to the data. We utilized ubiquitous and affordable computing and sensing technologies to prototype a device capable of acquiring such data. Its main components are a Raspberry Pi and sensors connected to it. Sensors included two distance sensors, a gyroscope, an accelerometer, a real-time clock, and a GPS antenna and receiver. For choosing each component’s brand and model, we considered cost, size, user-friendliness, availability, reliability, and applicability to our research’s main goal. Table 2 summarizes the chosen components and their model, manufacturer, and approximate retail price.

First, we chose to use a Raspberry Pi 4 (RPi) microcomputer to perform signal and data processing because of its low retail price, versatility, storage capability, and documentation availability. Moreover, its previous models have been proven to work successfully as data loggers for naturalistic cycling data collection [7,35,36]. The RPi is a single-board computer that can be used in a wide range of applications, from home projects like plant watering automation to commercial projects like cluster computing [37]. It has a powerful quad-core ARM Cortex-A72 processor, 8 GB of RAM, Gigabit Ethernet port, two USB 3.0 ports, two USB 2.0 ports, microSD card slot, and Bluetooth and Wi-Fi connectivity. The operating system running in each unit is the standard Raspbian, which supports a variety of programming languages (e.g., Python 2/3, C, C++, JavaScript, and others) [38].

For choosing the distance sensor, we compared RADAR, LiDAR, and ultrasonic sensor modules supported by the RPi. We opted to use ultrasonic sensors because of their cost–benefit analysis when considering the scope of this study. We considered the sensors’ cost and ability to measure distances over an appropriate range (i.e., a range covering at least the width of a typical traffic lane, about 3 m to 4 m). All of the distance sensors considered are capable of measuring distances within the appropriate range. LiDAR sensor modules had the widest distance range, but their cheapest version found at retail cost hundreds of dollars. While RADAR sensor modules for RPi can be found at a lower price than LiDAR sensor modules, they are scarcer and more expensive than ultrasonic sensor modules. Ultrasonic sensor modules are available for less than ten dollars per unit, which is in line with our research budget. In particular, we selected the HC-SR04 ultrasonic sensor module, which has been widely used as a distance measurement sensor [39,40,41] and does not require complex techniques to interface with the RPi [42].

**Figure 2:**
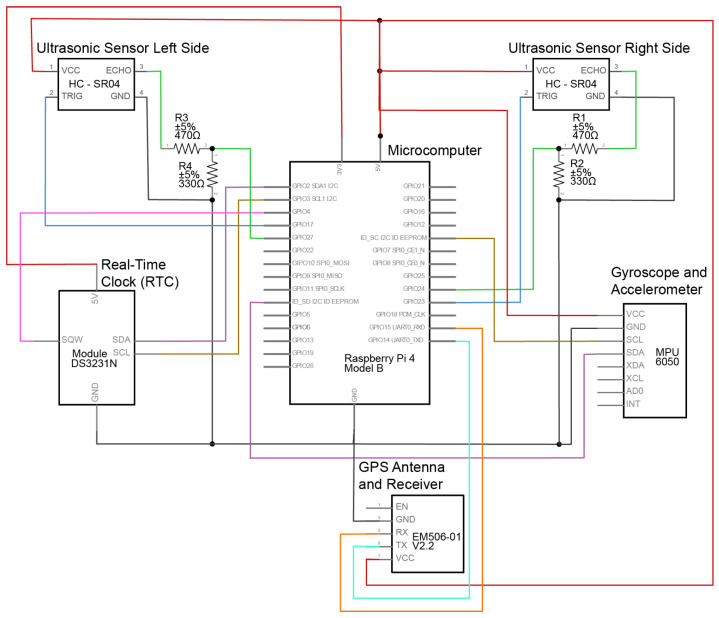
BSafe-360 Schema.

The connection between the ultrasonic sensors and the RPi is made through standard GPIO pins. Figure 2 shows the schema of the connection between the components and RPi. While these pins run at a voltage of 3.3 V, the HC-SR04 ultrasonic sensor module runs at a voltage of 5 V. Thus, resistors are added to lower the voltage. Equation (1) was used to determine the minimum resistance needed. Then, the resistance was distributed between Resistors 1 and 2 to guarantee that the input back to the sensor was recognized as high or low. The HC-SR04 module works by emitting a 40 Hz frequency ultrasonic wave from its transmitter that bounces back from obstacles within its range to the sensor’s receiver. Then, the ultrasonic sensor measures the time between the emission and reception of the waves and sends back a 5 V signal to the RPi. The HC-SR04 measurements range from 2 cm to 400 cm with an accuracy of ±3 mm. Although the measurement accuracy of the ultrasonic sensor can be affected by weather conditions, it would take severe conditions to alter the time between transmitted and received waves [42]. Data collected using BSafe-360 were for rides under mild weather conditions. Therefore, the measured distances are within the specifications of the HC-SR04 sensor.

(1) I=V/R,

where *I* is the current, *V* is the voltage, and *R* is the total resistance (R1+R2).

**Figure 3:**
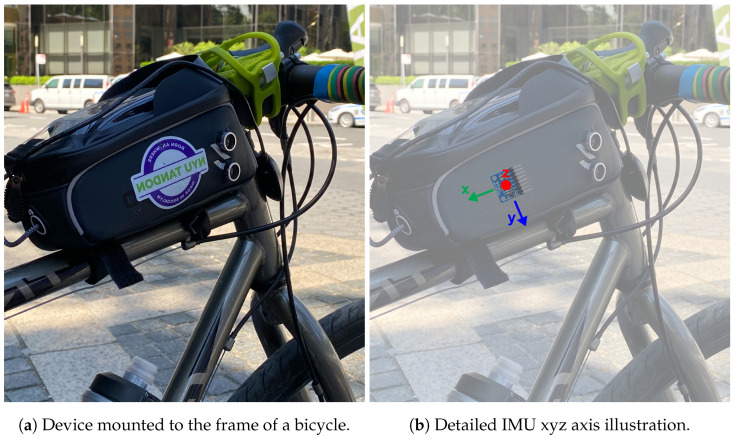
Photo of the BSafe-360 device mounted to a bicycle (**a**) and illustration of IMU xyz axis in reference to the device and bicycle (**b**).

The RTC chosen was the DS3231N module. The module interfaces with the RPi through an I2C connection and is powered by a coin battery when the RPi is off. I2C stands for Inter-Integrated Circuit, and it is a serial bus protocol used for avoiding data loss when allowing a faster device (RPi) to interface with a slower device (RTC) [43]. Another module that connects to the RPi through I2C is MPU6050, which has a three-axis accelerometer, a three-axis gyroscope, and temperature sensors. The accuracy ranges for the accelerometer and gyroscope are programmable by the user and can be ±2 g, ±4 g, ±8 g, and ±16 g and ±250${}^{\circ }$, ±500${}^{\circ }$, ±1000${}^{\circ }$, and ±2000${}^{\circ }$/s (DPS) [44], respectively. Here, g corresponds to gravity, and DPS corresponds to degrees per second. The accuracy ranges used in the BSafe-360 device are ±2 g for the accelerometer and ±250${}^{\circ }$ for the gyroscope. The temperature sensor ranges from −40 °C to 85 °C ± 1 °C. The three-axis configuration of the MPU6050 is detailed in Figure 3b. The x and y axis are opposite to the front direction of the bicycle and gravity because of space restrictions and the need to securely fix the MPU6050 to the enclosure securely using screws. However, the outcome data have the same quality as those of any other position.

Last, but not least, GlobalSAT BU-353-S4 was the chosen GPS receiver. This GPS receiver has an EM506-01 V2.2 module with a horizontal position accuracy of 2.5 m and a velocity accuracy of 0.01 m per second. It is noted in the manufacturer’s specifications that the time to first fix (i.e., when the GPS finds and fixes satellite signals when it is started) is 35 s for cold start (i.e., no previous fix recorded) and 1 s for hot start (i.e., the GPS still has information about available satellites stored in memory). The GPS receiver has a red light-emitting diode (LED) that blinks when the fix is found, which can be used by riders using the BSafe-360 device to confirm that the device is collecting GPS data before starting the ride. In practice, GPS can take up to 30 min to find its first fix in urban canyons. Within three days after the first fix, the GPS takes up to ten minutes to find the fix the next time it is turned on.

The assembled device, including its components and connections, is enclosed by an adapted bicycle phone case and mounted on the bicycle frame, as shown in Figure 3. The adaptations made to the case include opening holes on the left and right sides to fit the receivers and transmitters of the ultrasonic sensors and fixing all the components to the case using screws and nuts. An external dual USB power bank is used to supply power to the device, with its brand and model varying depending on availability at the unit’s building time. However, its lithium-ion battery has an energy capacity of 10,000–12,000 mAh and has a 5 VDC output. Each power bank can provide continuous power for approximately 10–15 h when the device is on standby and for up to 3.5 h when collecting data. Each assembled device also comes with a power cable with an on–off switch already connected to the power bank. Instructions can be provided to volunteers to use the switch to turn the BSafe-360 device on to start data collection and off to stop data collection. Volunteers can also troubleshoot powering issues by opening the case and removing or plugging the power cable from the power bank without having to interact with the RPi.

The combination of the data from these sensors provides us with data on the trajectory of the bicycle, the lateral distance between the bicycle and objects to the left and right sides, the 3D position (i.e., acceleration and angular velocity) of the bicycle throughout the trajectory, and the temperature inside the device. The next section describes the software development process for receiving the input signal from the sensors, processing them into the desired data, and storing the output data on easy-access databases.

#### 3.1.2. Software

We developed two Python 3.8 scripts to process and store the sensors’ raw data. Each script was divided into five main execution blocks:

1. Setup phase: when required libraries were imported.
2. Initialization phase: when the functions, constants, GPIO setup, and database connection required to process the sensor data were defined.
3. Processing phase: when the data from the sensors were acquired and processed by calling the predefined functions.
4. Storage phase: when the data was stored or displayed.
5. Termination phase: when the other processes were interrupted or stopped, and the database connection was closed.

The first script was responsible for triggering the sensors, processing their signal, and saving the data locally (i.e., signal processing and data logging). This script was named data_acquisition.py. The provided script (data_acquisition.py) is capable of achieving real-time synchronization of multi-sensor data. It effectively gathers data from all available sensors and consolidates them into a single record that includes comprehensive information for all variables of each sensor before sending them to the local and online databases. Therefore, each record in the data set exported to the databases is already unique and includes a timestamp that accurately reflects the moment the data was collected with all sensor features required for the data analysis step.

In the setup phase, libraries specific to each sensor were imported in addition to basic libraries. These specific libraries include mpu6050 and gps. The full list of libraries used for this script (data_acquisition.py) and the next one (load_postgresql.py) was made available in the project’s GitHub repository [45]. The initialization phase included defining the input and output GPIO for the ultrasonic sensors and MPU6050, the name of the log file, the types of log information, and the unit MAC address. It also included the definition of functions acquiring and processing the GPS and ultrasonic sensor data, logging all data, creating the local database connection, and defining the SQL queries used. These functions were then used in the processing and storage phases. The final output was a database file (connbike.db) with 24 features for each data point. The data acquisition script is set up to collect two records per second at temperatures specified by the manufacturers of all sensors used in BSafe-360 and in dry weather. Extreme heat or rain can reduce it to one record per second. However, it is possible to increase the sampling frequency to match the sensors’ data sampling frequency by reducing the time log variable in the script when a higher sampling rate is required. For example, one would need a higher sampling rate for the accelerometer and gyroscope data if they are interested in knowing the exact position and cycling behavior of the cyclist within the bicycle lane or when identifying irregularities in the pavement. The features and their descriptions are summarized in Table 3.

The second script (load_postgresql.py) checks for an internet connection, reads the data in the local database and sends the data to an online database hosted on a PostgreSQL server. The internet connection must be enabled and set up in the RPi for the script to be able to make the connection between the local and online databases. For the proof of concept presented in this paper, there was no internet connection throughout the rides. The RPi was set up to access the research center’s internet network. Therefore, the script connected to the center’s network and started the data transfer as soon as the device was on and back to the center’s building. However, it is also possible to set up the RPi to access the internet through the rider’s phone hotspot (i.e., a feature that lets the user share the cellular data connection of their phone with other devices) or through a cellular Subscriber Identity Module (SIM) card (i.e., a card used to enable communication with the device and a cellular network provider).

The most relevant library utilized, in addition to basic ones, was psycopg2, which allows the interface between Python and PostgreSQL. The initialization phase mainly consists of functions to establish the connections to local and online databases and queries. First, a copy of the original database file is created to avoid corruption of the file. Then, the internet access is verified, and connections are established between the copied file (local database) and the online database. The script checks which data were in the local database but not in the online one. In the storage phase, the script updates the online database with the current data that are missing. Last, the connections to both databases are closed.

In the spirit of open-source knowledge and collaborative research, all scripts and documentation were made available in the project’s GitHub repository [45] so that other researchers could use them to create their version of BSafe-360 or contribute to its improvement.

### 3.2. Data Analysis

#### 3.2.1. Data Processing

After the data are stored online, they can be accessed at any time for analysis by using the PostgreSQL user interface or by creating a Python script that uses the psycopg2 library to communicate with the database. Other languages or programs that connect to PostgreSQL can also be used instead of Python (e.g., R, Tableau, ArcGIS Pro, etc.).

**Figure 4:**
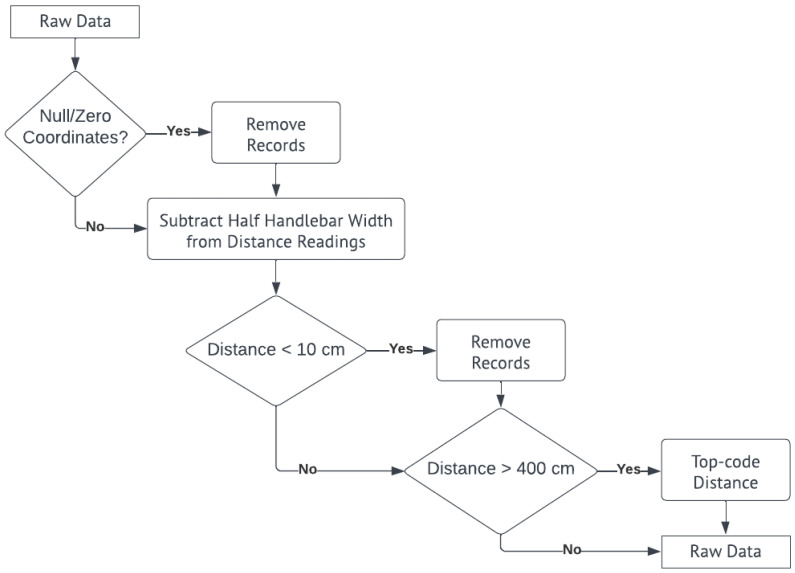
Data wrangling process.

We wrangled the raw data and obtained descriptive statistics in the same script using the Pandas library. In the wrangling stage, we eliminated null and zero latitude and longitude readings, then removed duplicates. We also used mapping tools (e.g., Tableau and ArcGIS Desktop) to remove the readings not corresponding to the rides (i.e., readings at home and readings before starting and after ending a ride). Half the width of the handlebar was subtracted from the total distance from each side to account for the positioning of the device on the frame (i.e., the middle of the bicycle). Then, we removed readings below 10 cm to account for possible erroneous readings. Finally, we top-coded the distance readings from both sides to 400 cm to account for the range of the ultrasonic sensor module. Any reading greater than 400 cm was interpreted as not a lateral obstacle of interest near the bicycle. Figure 4 summarizes the wrangling process described as a flowchart. The cleaned data can then be utilized for assessing bicycle safety through static or dynamic visualizations or more in-depth statistical analysis and modeling, as presented in Bernardes et al. (2023) [46].

#### 3.2.2. Data Visualization

**Figure 5:**
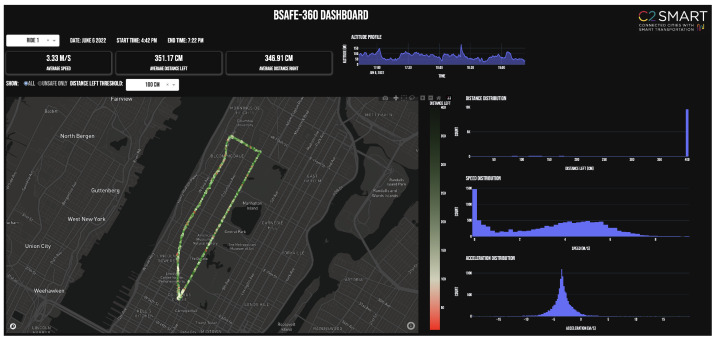
Dashboard for consolidating BSafe-360 data.

We created a dashboard to consolidate the data from the online database and show key insights from a safety perspective to provide a quick overview of the rides (Figure 5). The dashboard includes the distributions for LPD (left side) in cm, speed in m/s, and acceleration/deceleration in m/s${}^{2}$. The dashboard was developed using the Dash library in Python. In a separate script, the connection to the PostgreSQL database was defined. This connection script works as a module that can be imported into the dashboard script. The layout of the dashboard was defined using HTML and CSS commands, which the Dash recognizes for displaying the input data.

The following section presents a case study with naturalistic cycling data using BSafe-360 for a route in Manhattan, NYC, to exemplify how the data can be used to assess bicycle safety and to work as a proof of concept.

## 4. Case Study

As proof of concept, an experienced male cyclist collected seven hours of naturalistic cycling data during the summer on a route in NYC. These seven hours were collected in the course of three days, in the afternoon period, with an interval of about one week between them. Each day the volunteer rode for about 1.5 h to 2.5 h. The description of the route is provided in the following subsection. He cycled on a gravel bicycle with a BSafe-360 unit mounted to it. We decided to have only one volunteer to collect the proof-of-concept data to avoid possible bias caused by driver and cyclist behavior (e.g., different passing behavior from drivers due to the age or gender of the cyclist).

The Institutional Review Board (IRB) approved the experiment for human subject research under project number IRB-FY2019-3547, and the volunteer signed a standard consent form describing the details and implications of the experiment as a requirement of participation.

These details included minimal instructions on how the volunteer should ride so that we could guarantee that the data collected was, in fact, naturalistic. The volunteer was instructed to ride as usual and monitor whether body parts crossed the sensor, which may result in erroneous readings. We also required the use of a helmet for safety reasons.

### 4.1. Route Selection

**Figure 6:**
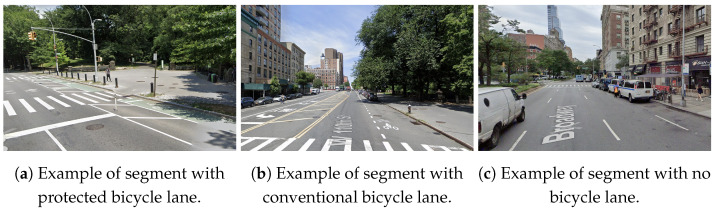
Photographs of segments of the route showing examples of infrastructure present in the route (source: Google Street View).

As part of the route selection process, we considered the time of completion, infrastructure, and accessibility. We chose a route that the average time to complete would be under an hour to ensure that we could collect data for multiple rides on each data collection day. We also ensured that the route had every type of bicycle infrastructure (non-existing, shared, protected) represented at least once on the route (Figure 6). To be more precise, the route consisted of 111 segments. Among these, 49 had protected bicycle lanes, 4 had regular bicycle lanes, 4 had shared bicycle lanes, and 54 did not have bicycle lanes. Finally, we ensured that the location of the route was easy to access for the rider using any mode of transportation but still in an area of interest for the scope of the research project.

**Figure 7:**
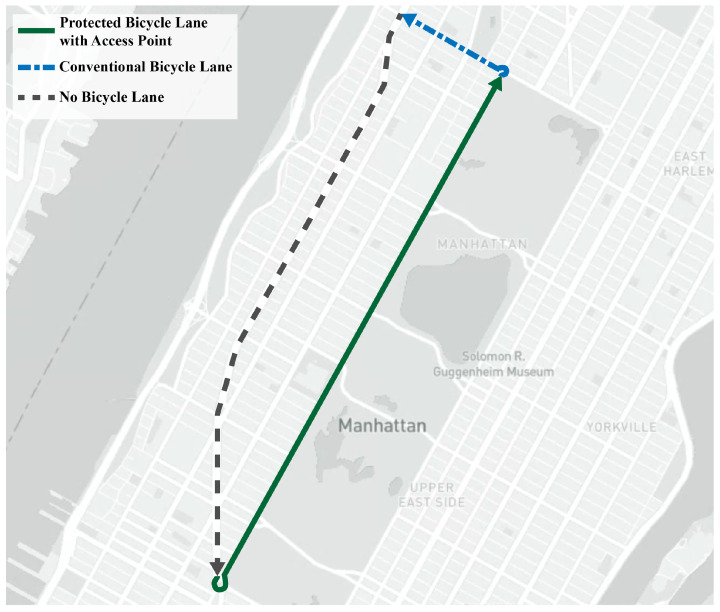
Route Map.

The selected route was 9.5 km long and was located in a mixed land use area in Manhattan, NYC (Figure 7). The area in which the route is located is known for heavy bicycle traffic, as the median percentage of people who ride a bicycle to work per census tract is 2.30%, which is higher than the percentage of people who ride a bicycle to work when considering the whole city (1.36%) [47].

### 4.2. Lateral Passing Distance

In bicycle safety studies, the LPD is defined as the distance between the bicycle and passing vehicles, and each vehicle that passes on the side of the cyclist is defined as a passing event. BSafe-360 is capable of collecting LPDs of passing events through ultrasonic sensors. For each passing event, the device registers one or more LPD readings, depending on how long the vehicle stays on the side of the bicycle. For example, if a speeding vehicle passes the cyclist, the device will record only one LPD reading for the passing event, whereas in the case where both the cyclist and the vehicle are slowly stopping as they approach an intersection, more than one LPD reading will correspond to the passing event. In this case study, we considered only readings from the ultrasonic sensor to the left side of the bicycle to be LPDs corresponding to passing events because that is the side on which the motor–vehicle traffic circulates. We also defined that only readings below 400 cm could indicate a possible passing event.

To check if the readings coming from the ultrasonic sensors were, in fact, from vehicles passing the bicycle, we simultaneously collected video data from NYC DOT’s closed-circuit television (CCTV) cameras [48] available throughout the route. CCTV is a system commonly used for surveillance and security, and its footage is monitored in real-time by either public or private agencies. Because the footage from these cameras is only available in real-time, we used the script developed by Zuo et al., 2020 [49], for collecting video data from 9 AM to 9 PM for all the days in which naturalistic cycling data were collected. The output data were screenshots obtained every 5 s. We collect data from the 11 cameras available on the route. However, some of the cameras did not face the route or had a poor resolution to identify the volunteer. In the end, we were able to check footage from six cameras, which were mostly in the same street.

**Figure 8:**
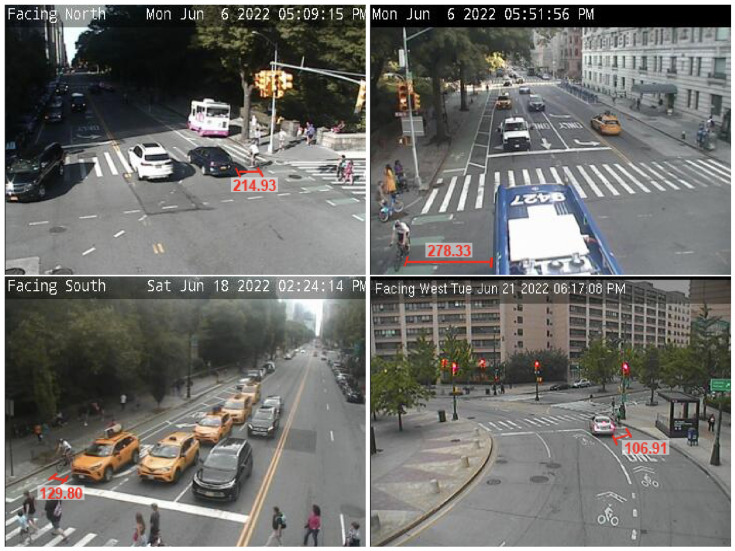
Examples of passing events identified and their LPDs labeled on screenshots of CCTV footage of the rides.

The process of checking whether the registered readings corresponded to vehicles overtaking the bicycle consisted of first noting the time and distance registered by BSafe-360 of data points with coordinates close to the camera locations. Then, we checked the screenshots from the CCTV cameras corresponding to the same timestamps and identified the volunteer based on their clothing, height, and skin color. We were able to identify the volunteer in about 18 screenshots. In seven of them, a vehicle was on the side of the volunteer, and we had a corresponding distance (Figure 8). In four screenshots, the volunteer was surrounded by other cyclists or by infrastructure, and readings below 400 cm were also registered. In the remaining screenshots, there was nothing by the side of the volunteer, and readings were 400 cm or higher. These results, combined with the previously mentioned testing of the ultrasonic sensors (Section 3.1) and the literature, show that the ultrasonic sensors in BSafe-360 are appropriate for measuring LPD. For example, Bernardes et al. (2023) [46] found an existing positive correlation between critical events collected by ultrasonic sensors and bicycle collisions in urban segments using Spearman rank coefficient.

It is worth noting that some researchers have opted for using action cameras to perform verification of passing events [25,30]. However, we opted to use CCTV rather than attaching an action camera to the bicycle because of budget, storage, and time constraints. For example, a GoPro camera with GPS capabilities costs more than $200.00, even the older models. Moreover, GoPro video files usually reach a size of more than 4 GB when recording for more than 10 min. The size of these files requires the acquisition of memory cards with large storage capacity, which adds up to the overall cost of the DAS. These files also require extra storage space in the computer, whereas the script used to collect CCTV data  [49] allows saving the video footage directly to the cloud or server. Finally, the use of video footage from action cameras leads to extra hours spent on uploading and preparing the files for data analysis.

### 4.3. Critical Events

As we are interested in critical events only, passing events with LPD readings higher than the threshold were not formally identified (i.e., LPD readings higher than 100 cm can correspond to a passing event or be part of a group of readings corresponding to a passing event). Critical events are defined as those in which the LPD is below a threshold. Many states in the U.S. define a safe LPD as 3 ft (∼100 cm). Thus, we consider a threshold of 100 cm for this study case. We calculated the total number of critical events by combining consecutive LPD readings as one event. Algorithm 1 shows the steps taken to define and identify critical events.
**Algorithm 1** Pseudo-code of the algorithm used to define critical events.  **for** each ride in rides **do**        **if** first record in ride **then**             **if** LPD ≤ threshold **then**                  $criticalevent_{1}\leftarrow 1$                  $eventid_{1}\leftarrow 1$                  eventidholder←1             **else**                  $criticalevent_{1}\leftarrow 0$                  $eventid_{1}\leftarrow 0$             **end if**        **else**             **for** each record in ride **do**                 **if** LPD ≤ threshold **then**                      $criticalevent_{record}\leftarrow 1$                      **if** $criticalevent_{record-1}=1$ **then**                            $eventid_{record}\leftarrow eventidholder$                      **else**                            $eventid_{record}\leftarrow eventidholder+1$                            eventidholder←eventidholder+1                      **end if**                 **else**                       $criticalevent_{record}\leftarrow 0$                       $criticalevent_{record}\leftarrow 0$                 **end if**             **end for**         **end if**  **end for**

### 4.4. Data Descriptives

After cleaning and filtering the raw data following the first steps of the wrangling process (Figure 4) presented in the data processing (Section 3.2.1) of the Materials and Methods section, we had 6 h, 35 min, and 56 s of data. The total number of valid LPD data points was 25,025, of which 946 were below the 100 cm threshold. These 946 data points resulted in 427 critical events. The duration of each loop of the route was, on average, 46 min.

**Figure 9:**
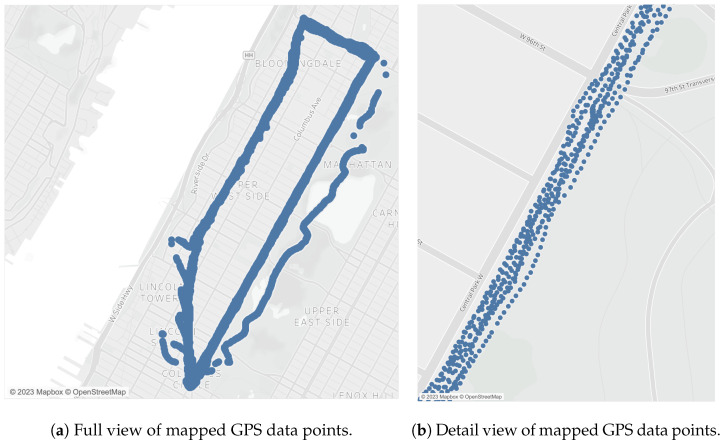
Mapped GPS data points in full view (**a**) and in detail (**b**).

The GPS data points were plotted using Tableau (Figure 9). When in a larger zoom, the accumulated points look like lines because of the small interval of up to one second between them. Although the precision of the GPS is 2.5 m, and the route is in an urban canyon, the data points properly matched the segments and intersections of the route, as confirmed by CCTV video footage. Data points far from matching the route were collected in rainy weather.

**Figure 10:**
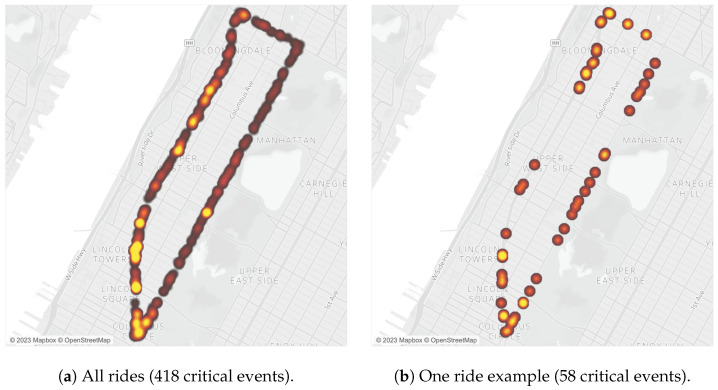
Heatmaps of critical events for all rides (**a**) and for a single ride (**b**).

After removing the data points that could not be visually matched to the route, we had 6 h, 4 min, and 14 s of data. The 23,959 data points resulted in 919 points with LPD of less than 100 cm and 418 critical events. Figure 10 shows heatmaps of the critical events observed for all rides and for a sample ride, which allows hotspot identification. We observed an average of 46 critical events per loop completed of the ride (s.t.d. = 27, min = 8, max = 88). As expected, due to them composing most of the route, non-existing infrastructure and protected bicycle lanes observed 56.70% and 33.89% of the data points, respectively. The conventional infrastructure portion of the route observed 7.09% of the data points, and the shared infrastructure portion observed 2.32%. A similar pattern was observed when looking into the critical events data. The portion of the route with non-existing infrastructure observed 54.07% critical events, the portion with protected bicycle lanes observed 38.76%, the portion with conventional bicycle lanes observed 6.22%, and the portion with shared bicycle lanes observed 0.96%.

**Figure 11:**
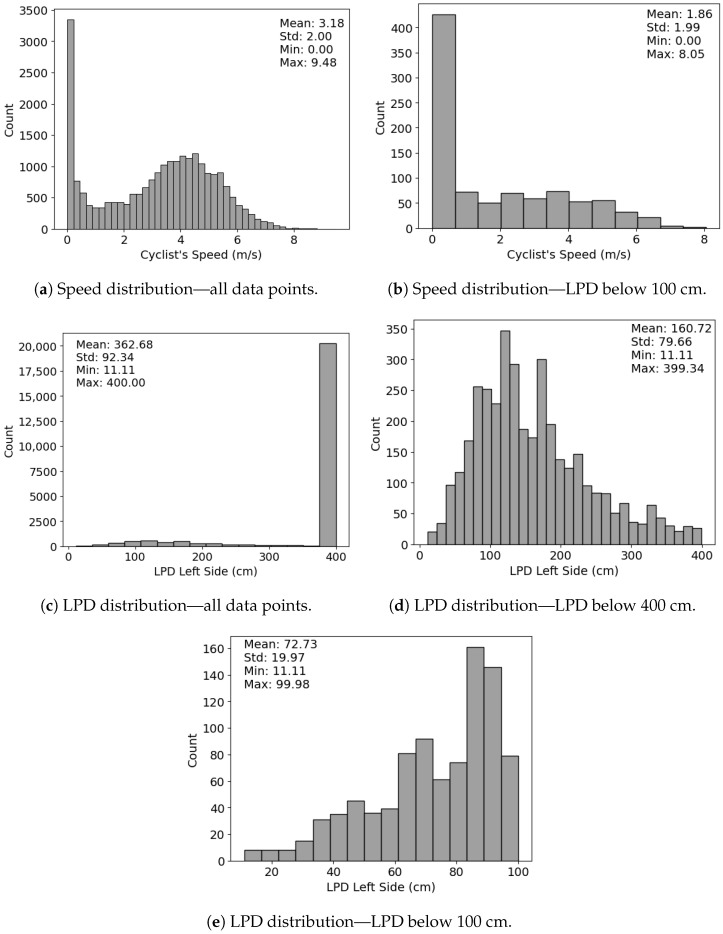
Speed distributions for all data points (**a**) and for data points with LPDs below 100 cm (**b**), and LPD distributions for all data points (**c**), for data points with LPDs below 400 cm (**d**), and for data points with LPDs below 400 cm (**e**).

Figure 11 allows comparing the speed and LPD distributions between all data points and data points with LPD below 100 cm. Figure 11a,b correspond to speed distributions, and Figure 11c–e correspond to LPD distributions.

**Figure 12:**
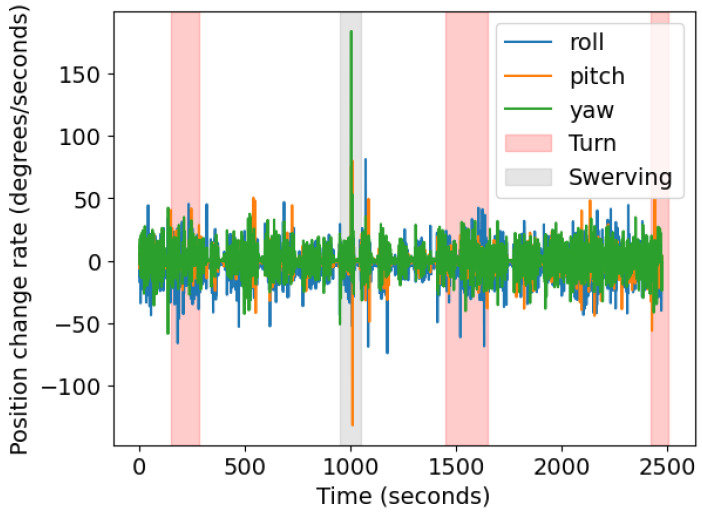
Gyroscope raw data for one sample ride.

**Figure 13:**
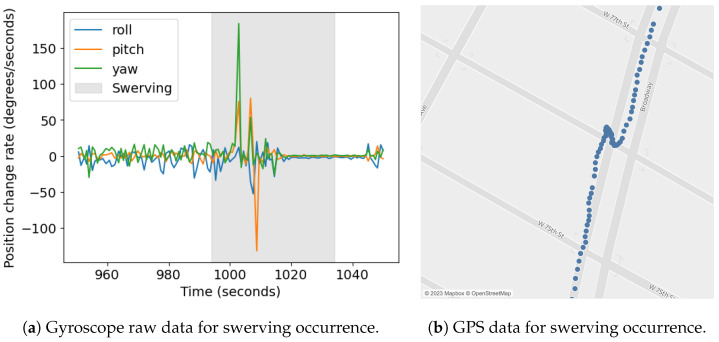
Gyroscope (**a**) and GPS (**b**) data for swerving occurrence.

Figure 12 shows the raw data for a sample ride with clearly marked turns and swerving events. The swerving event leads to a high peak in the gyroscope data, which is shown in greater detail in Figure 13a. The same swerving event could also be visually identified in the mapped GPS data (Figure 13b).

**Figure 14:**
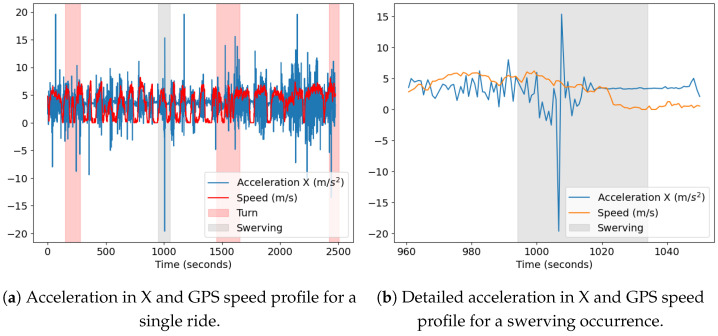
Acceleration in X and GPS speed profiles for one sample ride (**a**) and a detailed view for the same data profile for swerving occurrence (**b**).

Finally, Figure 14 shows the raw data from the accelerometer for the X vector and the GPS speed data. Although there is some noise in the gyroscope raw data, it is still possible to identify patterns in the data. For example, the speed and acceleration lines overlap and allow identification of the time when no acceleration is observed and when speed equals zero. The acceleration corresponding to zero speeds is close to the overall acceleration in X of 3.40 m/s${}^{2}$.

## 5. Discussion, Contributions and Limitations

The details of the development of the BSafe-360 DAS and the proof-of-concept case study presented in this paper demonstrate the device’s ability to collect naturalistic cycling data in a real-world setting, thus offering an efficient, reliable, and open-source tool to collect naturalistic cycling data for the academic community. The results demonstrated the feasibility of developing a data DAS that can successfully collect naturalistic cycling that includes crucial data such as LPD. The data collected using the BSafe-360 device provide valuable insights into the behavior and safety of cyclists. The heatmap of critical events identifies which segments of the road where vehicles overtake cyclists at an unsafe distance on the road and how often it happens. We observed many data points with speeds exactly or close to 0 m/s, which was expected due to the presence of several signalized intersections along the route. We also observed many LPD readings of 400 cm, indicating that no vehicle overtook the cyclist in the adjacent lane for most of the ride. Very few LPD readings were below 30 cm. These readings could be from body parts (e.g., knees or arms) crossing in front of the sensor due to the bad positioning of the cyclist on the bicycle. The gyroscope and acceleration data showed the potential to identify patterns of cyclist behavior. For example, we could identify a swerving event in the gyroscope data, also present in the mapped GPS data.

The introduction of BSafe-360 as a DAS to collect naturalistic cycling data provides a new opportunity for researchers and policymakers to improve the assessment of bicycle safety. In the last two decades, many methods have been introduced for collecting naturalistic cycling data, including GPS, video data, virtual reality, and multi-sensor platforms (e.g., instrumented bicycles and all-in-one mountable devices). However, most of these methods rely on syncing and interpolating the collected data from the sensors using a separate script or program, which can lead to noise or data loss.

BSafe-360 is unique in that it is an all-in-one platform, unlike most IBs or devices found in the literature [25,50]. Its ability to have multiple sensors in one DAS and combine their data in one data set is a major advantage over other existing methods of collecting naturalistic cycling data. For instance, virtual reality and multi-sensor approaches, such as IBs, have the advantage of high accuracy of commercial devices (e.g., Garmin’s bicycle computer, GoPro cameras, Velodyne LiDARs, etc.) but come with the challenge of data synchronization and cost of resources required. Each sensor generates a file, and these files need to be combined through data integration in post-processing. Such an approach increases the storage space required and possible errors due to the merging of data sets of sensors with varying frequencies.

Moreover, most commercial sensors have about the same or higher monetary cost as one unit of BSafe-360. Thus, combining several sensors to assemble one IB for real-world or virtual reality data collection can become extremely expensive. Even for low-cost DASs that do not use commercial sensors but require an IB-like assembly, such as the one proposed by Pérez-Zuriaga et al. (2022) [28] (∼€395.00 or ∼$425.00), cost more than BSafe-360 (∼$300.00) without accounting for the bicycle/e-scooter purchase. These costs also hinder the production of multiple units. BSafe-360 not only has a lower unit cost but also eliminates the need to acquire a bicycle/e-scooter for data collection. Researchers can opt to use the volunteers’ private bicycle/e-scooter or make use of existing bicycle-sharing systems (e.g., Citi Bike in NYC), as Bsafe-360 units can be easily mounted on any type of frame.

The BSafe-360 device has undergone several iterations since its first introduction by Bernardes, Kurkcu, and Ozbay (2019) [24] to achieve an easily scalable and reliable prototype. The software components were improved to bring the interval between observations from a range of 4–10 s to 0.5–1 s. The software also allows predefining an exact frequency within 0.5–1 s. The IMU unit was added to the original DAS to collect more key cycling data, and a regular phone case for bicycles replaced the 3D-printed case. We chose to alter the regular phone case instead of using a 3D-printed case because the monetary and time costs hinder the capability of having multiple units distributed to volunteers. Although 3D printing provides a custom enclosure and is the preference of other authors [23,25], the printing of one unit takes more than 10 h. whereas phone cases can be bought for cheaper when in bulk, and one unit can be adapted for fitting all sensors in less than 30 min.

The high portability of the prototype of the device allowed the units to be sent to various cities worldwide, including Denver in Colorado in the U.S., Brasilia in Brazil, and Shanghai in China, at different project stages (Table 4). Although prototype versions were sent to all three cities, some units were earlier versions than others. For example, the units sent to Denver and Shanghai in 2020 were missing a connection to the online PostgreSQL database and MAC address identification, which made it challenging to access the data collected in those cities immediately. As a result, there were some delays in comparing the data, minor miscommunication among the researchers involved, and a lack of standardization of the data analysis. Despite the lack of unique identifiers for those units, geographical coordinates could be used to address this issue.

**Figure 15:**
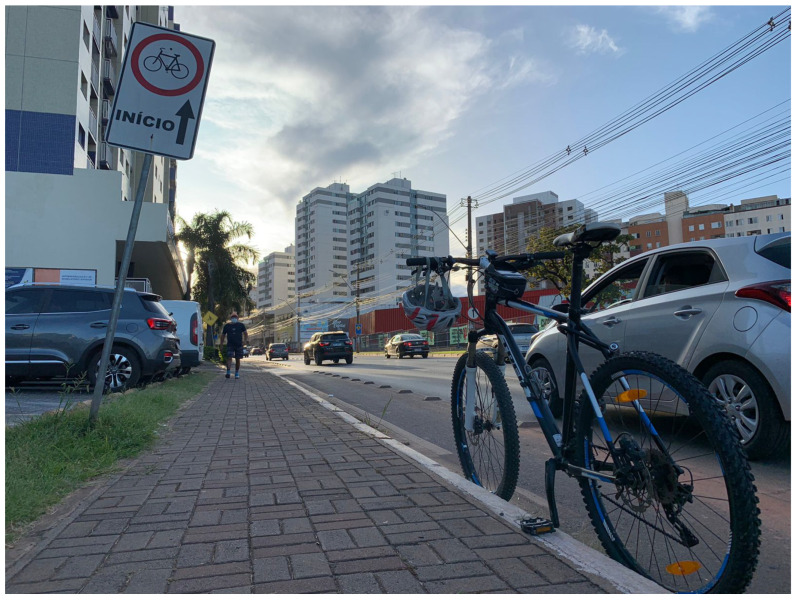
BSafe-360 being tested by researchers in Brasília, Brazil. Photo by: Marcel Mello.

On the other hand, the unit sent to Brasília in 2022 was closer to the current version of the device (Figure 15), with only missing improvements to the soldering and stabilization of components. Therefore, all the data collected in Brasília were readily available to all collaborators due to the addition of a PostgreSQL database connection, which avoided delays due to data transfer or miscommunications, proving that the use of an online database is an important feature to guarantee researchers access to reliable and standardized data independently of where in the world the study is being conducted. This is an important step to allow the comparison between bicycle safety studies and improve communication among the research community. The latest version of the device can be found in the project’s GitHub repository [45].

The use of single instruments (e.g., GPS or video footage) has limitations with respect to the accuracy and completeness of the data collected. Although it can provide the exact position of the cyclist within the traveling lane, it still needs other sensors to complement its data for bicycle safety. Even if using the GPS in smart devices (e.g., smartphones and smartwatches), which also have gyroscope and accelerometer sensors, crucial data about LPDs would be missing. Moreover, a high-precision GPS or a smart device alone is usually more expensive than BSafe-360. Regarding video footage, using video data from existing cameras in the infrastructure (e.g., CCTV cameras) can be limited by the camera’s direction variations and resolution, as evidenced by the case study. More reliable video data would require proper high-resolution cameras positioned high enough or drones. In the case of the camera, authorization from the city might be needed before installation, and in the case of the drone, some cities forbid their use altogether (e.g., NYC) [51]. BSafe-360 avoids such issues by collecting only anonymized data and not requiring its installation on public infrastructure.

In Table 5, we summarize the five all-in-one DASs found in the literature. It is worth noting that the documentation for the all-in-one DAS named OpenBikeSensor [32] is available mostly in German. Therefore, we used ChatGPT, an Artificial Intelligence tool, to translate details about its development to English to allow the comparison between the OpenBikeSensor DAS and the other DASs. All five DASs covered in the comparison follow a similar architecture. However, BSafe-360 and MetreBox present a higher portability than One Metre Plus (1M+) and PassBox due to their dimensions and placement on the bicycle. The placement on the frame and below the seat of the bicycle guarantees more stability to the BSafe-360, MetreBox, and PassBox devices, as the handlebar is constantly moving and can generate readings at varying angles throughout the route that might require further calibration. All four devices present reading ranges and frequencies suitable for bicycle safety studies, as they provide ranges large enough to cover a standard traffic lane and can collect readings with intervals of 1 s or less.

BSafe-360 has several advantages over the other three DASs. First, it has a combination of the existing features of the other four DASs in one device. It is more portable and easily customizable due to its size and the use of an adapted phone case. Additionally, it includes an IMU integrated into the device itself that provides important cycling behavior data such as pitch, roll, and yaw, which can be used for higher precision of bicycle positioning in the lane, identifying uneven road conditions and detection of collisions or falls. PassBox, the only other DAS that offers accelerometer and gyroscope data, requires the use of the IMU module of commercial action cameras, which increases the cost of the DAS by at least $200.00. The BSafe-360 device also uses real-time synchronization of sensor data to avoid post-processing errors.

Moreover, it stores data using both local and online databases. While the OpenBikeSensor also stores data locally and online, it requires users to create an account on their portal and actively upload the data into the portal database. These two extra steps might delay access to the data by the researchers, whereas BSafe-360 automatically uploads the data to the project’s online database as soon as internet connection is established. Finally, the use of a common bicycle phone case makes BSafe-360 the most discrete DAS, which helps avoid possible changes in cycling and driving behavior due to the cyclist’s and drivers’ awareness of the presence of the sensor. For future research, it would be interesting to reproduce all five DASs and collect data with them mounted on the same bicycle, as they can be placed in different places on the bicycle to compare their performance more accurately.

This study also allowed us to identify limitations of BSafe-360 that should be further researched. The device relies on ultrasonic sensors to collect LPDs, which may not be accurate under all conditions. For example, sensors may not accurately record distances in heavy rain, snow, or situations with a lot of background noise. This sensitivity to weather is also true for the GPS sensor. It presented inconsistency in the data under the rain, as observed in Figure 9. Additionally, the accuracy of the GPS can be affected by urban canyons, as observed in some parts of the route surrounded by tall buildings. The GPS limitations can be addressed by applying the Kalman Filter or Dead-Reckoning methods to predict the exact trajectory in real-time. The position of the IMU at a slight angle resulted in a 3.4 m/s ${}^{2}$ offset from zero for the acceleration readings in the x-direction. Therefore, we suggest that further calibration must be performed for future studies to take into account the angle of the bicycle frame in relation to the pavement.

Finally, although we collected data from multiple users using BSafe-360 for other purposes, we included data of a unique rider in this paper to ensure that different rider behaviors did not bias the validation of the DAS. By gathering information from a single cyclist, we were able to comprehend the fundamental operations of the DAS. This enabled us to demonstrate the feasibility of BSafe-360 as a comprehensive all-in-one naturalistic cycling data collection tool. However, we suggest the collection of data from cyclists of different backgrounds (e.g., gender, cycling experience, and age) for more complex studies that make inferences about the impact of drivers, cyclists, and infrastructure variables on bicycle safety. Therefore, more studies are required to validate the robustness and generalizability of the DAS in different contexts and evaluate its impact on the safety of cyclists.

## 6. Conclusions

This paper introduced a DAS called BSafe-360 for collecting naturalistic cycling data that is efficient and reliable. The results of this study show that BSafe-360 makes a significant contribution to the field of naturalistic cycling data collection. It addresses the limitations of existing methods by providing an all-in-one DAS that combines multiple sensors in a single device. This unique feature eliminates the need for data synchronization and interpolation, ensuring the accuracy and completeness of the collected data. BSafe-360 allows the collection of data such as the trajectory of the cyclist, LPDs, acceleration, pitch, roll, and yaw, which are crucial for bicycle safety studies by providing valuable insights into cycling behavior, interactions between cyclists and other road users, and infrastructure.

The BSafe-360 device’s high portability and scalability enable widespread deployment, which has the potential to improve large-scale bicycle studies and data collection worldwide significantly. Another advantage of its portability is that it is ready to be used without the need for professional assembly, not only in traditional bicycles but also in most micromobility vehicles such as e-scooters and electric bicycles. By offering an efficient and reliable means of collecting naturalistic cycling data, the BSafe-360 system enhances the assessment of bicycle safety and supports evidence-based decision-making for policymakers, urban planners, and researchers in the field.

The proposed DAS has been proven to be a highly versatile instrument that surpasses its original safety focus. Its efficient data collection abilities render it capable of monitoring a variety of infrastructure types and even functioning as an advanced bicycle computer. The presence of a second ultrasonic sensor measuring the lateral distance from the right side of the bicycle allows the easier adaptation of the device to different traffic configurations in cities in several countries. For example, in some streets of Brasília in Brazil, the traffic is to the right side of the cyclists (Figure 15). Moreover, the availability of distance data from both sides of the bicycle can provide a better understanding of the position of the cyclist in the bicycle lane or the traffic lane.

Finally, readings from the right side of the bicycle can help understand the impact of parking, double parking or exits on bicycle safety. With the addition of a user-friendly touch-screen display, BSafe-360 could provide cyclists with a comprehensive range of information (e.g., safety alarm indicating the presence of an overtaking vehicle below safe distance, bicycle speed, options of safer routes, etc) which could help cyclists make better decisions regarding their ride and safety in real-time. Additionally, if equipped with a camera, BSafe-360 could accurately identify and calculate the speed of overtaking vehicles, which could contribute even further to advancing bicycle safety studies by better characterizing vehicles that contribute the most to bicycle collisions, injuries, and fatalities.

Furthermore, the BSafe-360 wireless capabilities enable it to seamlessly connect with other vehicles, infrastructure, and smart devices, unlocking a plethora of exciting new possibilities (e.g., alerting drivers of dangerously close bicycles, alerting first responders to possible bicycle collision, mapping road conditions, etc.), which could help cyclists make better decisions regarding their ride and safety in real-time. Finally, BSafe-360 can be easily reproduced and improved by other researchers for their studies, as its components information and hardware schema was made available in this paper, and its scripts were made available in the GitHub repository [45]. Overall, BSafe-360 is an asset that can serve a wide range of purposes, making it a valuable tool for the advancement of bicycle safety and various other industries.

## Figures and Tables

**Table 1:** Summary major studies in DAS for bicycle and other micromobility studies.

Study	DAS	Lateral Distance Measurement Technology	Micromobility Vehicle Used	Problem of Interest
Pérez-Zuriaga et al. (2022) [28]	IB	Ultrasonic Sensors	E-scooters	Rider Comfort
Nolan et al. (2021) [30]	All-in-one	Ultrasonic Sensors	Traditional Bicycles	Rider Safety
Ma et al. (2021) [29]	IB	-	E-scooters	Rider Comfort
Henao et al. (2021) [25]	All-in-one	Ultrasonic Sensors	Traditional Bicycles	Rider Safety
Dozza, Bianchi Piccinini, Werneke (2017) [33]	IB	-	Electric Bicycles	Rider Safety
Bernardes, Kurkcu, and Ozbay (2019) [24]	All-in-one	Ultrasonic Sensors	Traditional Bicycles	Rider Safety
Beck et al. (2019) [23]	All-in-one	Ultrasonic Sensor	Traditional Bicycles	Rider Safety
Jeon and Rajamani (2018) [8]	IB	LiDAR	Traditional Bicycles	Rider Safety
Dozza, Rasch, and Boda (2017) [7]	IB	-	Traditional Bicycles	Rider Safety
Dozza and Werneke (2014) [6]	IB	-	Traditional Bicycles	Rider Safety
Joo and Oh (2013) [4]	IB	-	Traditional Bicycles	Road Conditions

**Table 2:** Component list.

Component	Model	Manufacturer	Approximate Price
Raspberry Pi 4 Starter Kit (Includes SanDisk 32 GB Class 10 MicroSD Card)	Model 4 8 GB	Raspberry Pi Foundation (kit from CanaKit)	$140.00
GPS	BU-353-S4-5 Hz	GlobalSat	$95.00
Ultrasonic Sensor	HC-SR04	ELEGOO	$2.00
3-axis Gyroscope and 3-axis Accelerator	MPU6050	SunFounder	$10.00
RTC	DS3231	HiLetgo	$4.00
Portable Charger	-	Various	$25.00
Enclosure	-	Various	$12.00
		Total	$300.00 ^1^

^1^ Total cost considering miscellaneous materials such as jumping wires, soldering, shrinking tubes, and screws.

**Table 3:** Feature list.

Source	Feature	Description
System	mac	Unique MAC address of the unit.
RTC	dtg	Timestamp without timezone.
GPS	latitude and longitude	Geographical Coordinates.
	timeutc and timefix	Timestamp in UTC and fixed.
	altitude	Altitude in meters.
	ept, epx, epv, and eps	Error estimates for timestamp (in seconds), longitude (in meters), altitude (in meters), and speed (in meters/seconds), respectively.
	speed	Speed in meters/seconds.
	climb	Rate of ascent/descent in meters/seconds.
	track	Heading in degrees from true north.
	mode	NMEA mode.
Left Ultrasonic Sensor	usreading_l	Distance from left side in centimeters.
Right Ultrasonic Sensor	usreading_r	Distance from right side in centimeters.
MPU6050	acce_x, acce_y, acce_z	Acceleration for x, y, and z vectors as function of gravity.
	gyro_x, gyro_y, gyro_z	Angular velocity in degrees/seconds.
	temp	Temperature in Celsius.

**Table 4:** Cities where researchers received BSafe-360 for collaboration.

City	Year	BSafe-360 Version	Number of Units Sent	Type of Bicycles Tested
Brasília, Brazil	2022	Present version	2	Private: traditional bicycles
Denver, Colorado, the U.S.	2021	Bernardes, Kurkcu, and Ozbay [24]	2	Private: traditional bicycles
Shanghai, China	2020	Bernardes, Kurkcu, and Ozbay [24]	2	Bicycle-share system: traditional bicycles

**Table 5:** Comparison of existing all-in-one DASs.

	Feature	BSafe-360	MetreBox [23]	One Metre Plus (1M+) [25]	PassBox [30]	OpenBikeSensor [32]
Hardware	Case	Adapted from commercial bicycle phone case	3D-Printed	3D-Printed	Plastic commercial project box	3D-Printed
	Distance Sensor	Ultrasonic Sensors (HC-SR04)	Ultrasonic Sensors (XL-MaxSonar-EZ3 MB1230, Maxbotix, Minnesota, MN, USA)	Time of Flight LiDAR (Tfmini plus micro lidar)	Ultrasonic Sensors (MaxBotix MB1232 I2CXL-MaxSonar-EZ3)	Ultrasonic Sensors (JSN-SR04T)
	IMU	MPU6050	Not present	Not present	IMU from Garmin Virb X	Not Present
	Placement	Frame	Below seat	Handlebar	Bicycle rack or seat post	Bicycle rack or seat post or handlebar
	Portability	High	High	Medium	Medium	Medium
Software	Data Storage	Local (SQLite) and Online (PostgreSQL)	Local (File format not disclosed)	Local (CSV)	Local (File format not disclosed)	Local (CSV) and Online (OpenBikeSensor Portal)
	Open-source	Yes	No	Yes	No	Yes
	Data Synchronization	Real-time	Post-processing	Post-processing	Post-processing	Real-time
Distance Readings	Frequency (Hz)	40	10	60	40	40
	Range (cm)	2–400	3–330	10–1200	20–765	20–600
	Side of Bicycle	Left and Right	Left	Left	Left and Right	Left and Right

## Data Availability

The data can be requested directly to the corresponding author.
